# Tactile facilitation during actual and mere expectation of object reception

**DOI:** 10.1038/s41598-022-22133-z

**Published:** 2022-10-20

**Authors:** Damian M. Manzone, Luc Tremblay, Romeo Chua

**Affiliations:** 1 Perceptual Motor Behaviour Laboratory, Centre for Motor Control, Faculty of Kinesiology and Physical Education, University of Toronto, 55 Harbord Street, Toronto, ON M5S 2W6 Canada; 2 School of Kinesiology, University of British Columbia, Vancouver, BC Canada

**Keywords:** Motor control, Sensorimotor processing, Sensory processing, Somatosensory system, Human behaviour

## Abstract

During reaching and grasping movements tactile processing is typically suppressed. However, during a reception or catching task, the object can still be acquired but without suppressive processes related to movement execution. Rather, tactile information may be facilitated as the object approaches in anticipation of object contact and the utilization of tactile feedback. Therefore, the current study investigated tactile processing during a reception task. Participants sat with their upper limb still as an object travelled to and contacted their fingers. At different points along the object’s trajectory and prior to contact, participants were asked to detect tactile stimuli delivered to their index finger. To understand if the expectation of object contact contributed to any modulation in tactile processing, the object stopped prematurely on 20% of trials. Compared to a pre-object movement baseline, relative perceptual thresholds were decreased throughout the object’s trajectory, and even when the object stopped prematurely. Further, there was no evidence for modulation when the stimulus was presented shortly before object contact. The former results suggest that tactile processing is facilitated as an object approaches an individual’s hand. As well, we purport that the expectation of tactile feedback drives this modulation. Finally, the latter results suggest that peripheral masking may have reduced/abolished any facilitation.

## Introduction

The perception of tactile events is reduced before or during action (i.e., tactile suppression^1^). Further, this reduced perception is evident for a range of movements, from simple finger abduction^2,3^ to more complex reaching and grasping^4,5^. Importantly, during a reaching and grasping movement, the individual’s goal is to acquire the target object (e.g., grabbing a stationary cup or ball). But we can also consider a situation in which individuals can still acquire the target object without executing a reaching movement. For example, when receiving or catching an object, the individual can grasp the target while keeping their limb still. Critically, the processing of tactile information during reception tasks may not be influenced, or influenced to a lesser extent, by the suppression processes evident during the execution of the reaching movement. Although the factors that influence tactile suppression have been broadly investigated^1,6^, less is known about tactile processing during a reception task and is thus the purpose of the current study.

Movement related tactile suppression likely stems from both central predictive and peripheral mechanisms, which are further corroborated by neurophysiological evidence of sensory attenuation along the dorsal column medial lemniscal pathway ^7–9^ (see also Ref.^10^). Specifically, internal forward models can utilize a copy of the motor command to attenuate expected sensory signals, which in the context of the current work, relates to reduced perception of tactile stimuli^11–14^. It is also important to consider peripheral accounts, which suggest that stronger afferent stimuli mask the perception of weaker afferent stimuli, even if the weaker stimuli are presented first (i.e., backward masking^15^). In the context of tactile suppression, movement-related afferent stimuli can mask the perception of the weak tactile stimuli presented to participants, even if the tactile stimuli are presented prior to movement onset^3^. Further, reduced perception^3,5^ and somatosensory evoked potential amplitude during passive movements^16,17^ provide evidence for peripheral masking (cf.^18^).

An important modulating factor when considering tactile processing during movement relates to the relevance of tactile feedback for the task at hand. For example, during a reaching and grasping movement, tactile feedback at the finger is relevant and utilized for the grasping portion of the task^19^. In the context of tactile suppression, there is evidence for a reduced magnitude of suppression at a task-relevant location (i.e., index finger) compared to an irrelevant location (i.e., forearm^5^; see also Refs.^20,21^). Further, the magnitude of suppression at the index finger is reduced when the task involves object contact, and therefore the utilization of tactile feedback, compared to a task that does not (i.e., pantomime reaching^5^). Thus, the central nervous system can flexibly modulate the processing of tactile events during movement, likely via the involvement of the dorsolateral prefrontal cortex^22–26^.

In some contexts, tactile processing is not suppressed and there is even evidence for facilitation rather than suppression. For example, perception rates were not found to be reduced when performing movements under 200 mm/s^27^. Such movement speeds are typically associated with exploratory actions in which the goal is to uptake tactile cues (e.g., braille reading^28^; see also Refs.^29,30^). This evidence is corroborated by enhanced perceptual performance when completing exploratory compared to reaching movements^31^. Further, when reaching to one’s own opposite hand without visual information, tactile perceptual thresholds are reduced on the target hand (i.e., tactile enhancement), and suppression can be decreased or eliminated on the moving hand along its trajectory^24,32,33^. Because the hand is the target of the reach, somatosensory information from the target hand is relevant to signal and update the target location throughout the movement, especially without vision of the target hand^33^ (see also Refs.^34–36^).

During a reception or catching task, an individual’s hand is contacted by an external object (e.g., ball). In these scenarios, individuals can use visual information to predict when the object will make contact with their hand^37–40^. Further, tactile feedback is critical for task success, to determine whether an object was received or not and for regulating the grip^19^. Thus, individuals can anticipate object contact and the subsequent utilization of tactile information for the task. If the individual is not moving toward the object immediately before or at the moment of reception/object contact, then tactile processing should not be influenced by the central and peripheral processes that contribute to the suppression of tactile information. Rather, tactile processing may be facilitated as the object approaches the individual in anticipation of the utilization of tactile feedback (see Ref.^41^ for an example of somatosensory facilitation). However, literature employing catching tasks have failed to find evidence for tactile facilitation^42,43^. But, in these studies, tactile perception was measured during movement components of a juggling task^42^ or when individuals were in the process of moving their limb to catch an incoming basketball^43^. Thus, central and peripheral suppression processes related to movement could have masked any facilitation processes.

The current study investigated tactile processing during an object reception task, and critically, individuals did not perform a reaching movement toward the object. That is, participants kept their limb still and were asked to detect tactile stimuli delivered during different points along the object’s trajectory and prior to contact. Our main hypothesis was that tactile processing would be facilitated rather than suppressed as the object approached the individual. Alternatively, the prediction of the collision between the object and the hand, and its associated sensory consequences, may cause suppression or a lack of enhancement of tactile processing. However, this explanation is less likely as recent evidence suggests that the generation of an efference copy via active movement is fundamental to sensory attenuation processes (cf. passive or no movement^44^). Rather, another key hypothesis was that the prediction of object contact and utilization of tactile feedback drives the facilitation of tactile processing. To test this hypothesis, we included a condition in which the object stopped early and did not contact the individual on only 20% of trials. Due to the high probability of contact (i.e., 80%; see Ref.^12^ for similar probability ratios), individuals should expect contact on every trial, even on the no-contact trials. Thus, if tactile processing is facilitated in this no-contact condition, this would suggest that the anticipation of object contact, and utilization of tactile feedback plays a crucial role for the modulation of tactile processing during reception. Further, we also investigated whether tactile processing was modulated based on the time/distance of the object relative to the hand. During the execution of goal-directed reaching, there is evidence for differences in sensory processing along the movement trajectory^21,33,45^. But this modulation may be specific to movement execution as sensory feedback can be differentially utilized along the trajectory to guide the movement toward the target^33,45^. Therefore, time/ distance-based modulation may not be expected during a reception task as no goal-directed reaching is required. In contrast, tactile processing may be increased when the object is closer to the individual’s hand compared to early in the object’s trajectory, as object contact and the subsequent utilization of tactile feedback will occur sooner. Our last hypothesis regarded the potential for backward masking when the participants are presented with a tactile stimulus in close temporal and spatial proximity with object contact. That is, if the stimulus is presented late in the object’s trajectory, and in close temporal and spatial proximity to the participant, the afferent information arising from object contact may mask the perception of the weaker tactile stimulus presented to participants. Thus, we hypothesized that any facilitatory processes when the stimulus is present late in the trajectory may conflict with backward masking processes and thus yield no systematic difference in individuals perception compared to a baseline condition (i.e., no reliable facilitation or suppression effect).

## Methods

### Participants

Eighteen participants (6 male, 12 female, mean age: 24 years old), with no self-reported history of neurological impairment completed the experiment. All but one participant self-reported as right hand dominant. Sample size was first estimated at 15 using G*Power with the effect size calculated from the results of Ref.^46^ (Cohen’s d = 0.79), beta set to above 0.8 and alpha set to below 0.05. Then, due to the novelty of the experimental protocol, we recruited the higher sample sizes in comparable studies (e.g., Refs.^24,32,46^), which also help deal with attrition. Indeed, two participants were excluded from statistical analyses because they detected the weakest stimulus intensity (i.e., 6 steps below the estimated threshold) 50% of the time. This stimulus intensity issue indicated that our initial threshold estimate was too high, and that these low detection rates may bias the calculation of detection threshold and variability. Therefore, sixteen participants (5 male, 11 female, mean age: 24 years old) were included in the statistical analyses. All participants provided written informed consent prior to their participation. The procedures of the study were approved by the UBC Behavioural Research Ethics Board and complied with the ethical standards of the 2013 Declaration of Helsinki regarding the treatment of human participants in research^47^.

### Materials, apparatus, and stimuli

**Figure 1:**
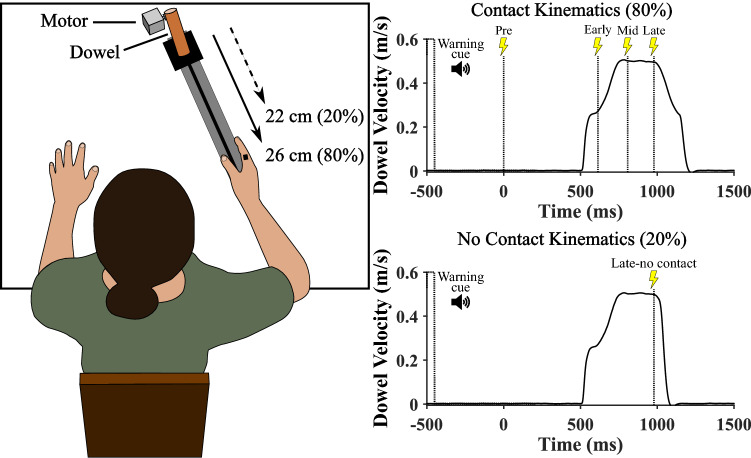
Left Panel: Illustration of the experimental setup. On 80% of the trials the dowel moved 26 cm (i.e., Pre, Early, Middle and Late stimulation trials) and on 20% of the trials the dowel moved 22 cm (i.e., Late-no contact stimulation trials). The stimulus location is depicted on the right index finger in black. Note that electrodes were placed on the dorsal and palmar surface of the base of the index finger. Right Panel: Dowel velocity profiles and stimulation times when the object travelled 26 cm and made contact with the individual (i.e., 80% of trials; top graph) and when the object travelled 22 cm and did not make contact with the individual (i.e., 20% of trials; bottom graph). In all conditions, a 50 ms auditory stimulus served as a warning cue and only one stimulus was presented for each trial. Note that the only difference between the contact and no contact kinematics was the sudden stop and sharp drop off in dowel velocity in the no contact dowel velocity profile.

Participants sat at a table with their right upper limb resting on the surface. Participants adjusted the placement of their arm such that a plastic dowel (~ 34 mm in diameter) was located 26 cm away from the edge of their hand when it was in a receiving posture (see Fig. 1). This was done in an attempt to synchronize the object stopping and the object contacting the participant’s fingers and avoid any significant skin stretching displacement of the fingers caused by the object. The dowel was placed on top of a carriage on a custom-built linear slide which was attached to a closed-loop belt controlled by a computer-controlled stepper motor (57HS82-3004 Nema 23 stepper motor driven by a JK0220 Microstep Driver). A pair of surface electrodes (i.e., anode and cathode; Kendall-LTP Soft-E Cloth electrodes) were taped to the dorsal and palmar surface of the proximal phalanx of the right index finger. The tactile stimulus was a 2 ms constant current pulse, generated by sending a 2 ms square wave voltage waveform to an isolated constant current stimulator (BIOPAC systems Inc., STMISOLA set at 1 mA/V). The intensity of the tactile stimulus was determined by the amplitude of the square wave voltage pulse (see “Procedure” section for stimulus intensities).

One infrared light emitting diode (IRED) was taped to the dowel to track the movement of the object. Further, participants were instructed to remain still throughout the trial, which was confirmed by IREDs placed on the index finger and thumb. The IREDs were sampled at 500 Hz for up to 2.5 s (Optotrak 3020, Northern Digital Inc., Waterloo, ON, Canada). The recording window was initiated approximately 100 ms before the onset of the warning stimulus (piezoelectric buzzer; 50 ms tone) and approximately 1000 ms prior to the onset of object movement. This recording window was chosen to have a record of all IRED positions for the duration of the entire trial. Custom written MATLAB (The MathWorks Inc., Natick, MA, USA) and LabView (National Instruments Inc., Austin, TX, USA) scripts were used to control the Optotrak and to generate the warning cue, a National Instruments NI-USB6002 Multi-function device was used to generate the analog waveform for the stimulator, and an Arduino Mega 2560 microcontroller was used to control the stepper-motor.

### Procedure

A participant was first presented with a stimulus that was assumed to be above their tactile threshold (i.e., 5 mA for 2 ms using the BIOPAC STMISOLA test pulse function). The next phase of the experiment included a staircase procedure, which was used to estimate each participant’s perceptual threshold. That is, participants were presented with stimuli that got gradually weaker until we reached an amplitude that participants felt about half of the time (i.e., initial stimulus amplitude: 2 mA; step size: 0.1 mA). This estimated threshold was used to create a custom range of amplitudes surrounding each participant’s initial threshold estimate. The range included the initial threshold estimate (i.e., step 0) plus 4 equal steps both above and below the estimate. In addition, 2 steps equivalent to 6 steps above and below the estimate were included. Therefore, there were 11 stimulus amplitudes in total (i.e., ± 6, ± 4, ± 3, ± 2, ± 1 and 0) with the step size equal to 0.07 mA. To limit the number of trials, there were 4 trials for steps ± 6, 5 trials for steps ± 4 and ± 3, and 8 trials for steps ± 2, ± 1 and 0. The fewer trials at the ends of the range were used to confirm the presence of the percept (or lack thereof). And the trials surrounding step 0 allowing for a robust estimate of perceptual thresholds (see Ref.^46^ for similar trial structure). Therefore, for each condition there were 68 trials. In addition, a total of 20 no-stimulation/catch trials were included to ensure that participants did not experience false alarms (i.e., performance on catch trials: M = 0%, SD = 0%). In total, participants completed 360 trials (i.e., 68 trials × 5 conditions = 340 trials + 20 catch trials).

On every trial, a warning cue was followed by the movement onset of the dowel approximately 1000 ms later. On 80% of the trials, a stimulus could be presented ~ 500 ms before the object started moving (Pre object movement condition) or at one of three distances/times during object motion. Presenting stimuli at different distances/times during object motion allowed us to investigate whether the distance/time of the object from the hand influenced tactile processing. In an Early condition, the stimulation was presented ~ 2.8 cm or 114 ms after object movement onset. In a Middle condition, the stimulation was presented ~ 11.4 cm or 308 ms after object movement onset. In a Late condition, the stimulation was presented ~ 19.9 cm or 478 ms after object movement onset. Note that only the distance between the stimulation conditions is approximately equal (i.e., same number of motor steps) because the object’s velocity profile was not linear (see Fig. 1). For these trials, the dowel moved 26 cm, the movement lasted ~ 672 ms, and the object made contact with the participant’s index finger and thumb. The timing of the Early condition is similar to that of previous work presenting a stimulation around 100 ms after movement onset^5^. Further, because there is evidence for decreased perception around 100 ms prior to a masking stimulus^3^, the Late condition increased the amount of time between the stimulation and object contact (i.e., 194 ms). On 20% of the trials, the dowel abruptly stopped after 22 cm, the movement only lasted 552 ms, and the object did not make contact with the participant’s index finger or thumb (see Fig. 1). On these trials, a stimulus was presented ~ 19.9 cm or 478 ms after movement onset and was considered the Late-no contact (Late-nc) condition. The only difference between the Late and Late-nc condition was that the dowel stopped early in the Late-nc condition and participants did not receive nor make contact with the object. At the end of every trial, participants verbally reported whether or not they felt the stimulus. For every participant, the combination of stimulation time (i.e., Pre, Early, Middle, Late, and Late-nc) and stimulus amplitude (i.e., from − 6 to + 6 including catch trials) was randomized.

### Data analysis

#### Finger stability

In order to confirm that participants remained still surrounding the stimulation time, the displacement of the index finger in the x (i.e., mediolateral) and y (i.e., anteroposterior) axes was measured during a 200 ms window, centered on the stimulation times. In the x axis, the maximum absolute movement distance was 0.78 mm (M = 0.09 mm). In the y axis, the maximum absolute movement distance was 1.13 mm (M = 0.25 mm). Therefore, the highest average velocity was below 6 mm/s surrounding the stimulation time with a mean of around 1 mm/s.

#### Perception

To calculate detection thresholds and the variability of stimulus detectability, we calculated the proportion of stimuli detected at each stimulus amplitude for all stimulation times. Then, the proportions for each stimulation time were fitted to separate psychometric curves with a logistic sigmoid using Psignifit 4.0 in Matlab^48^ (i.e., five curves per participant; see Supplementary Fig. S1 for individual participants curves). Detection thresholds were defined as the stimulus amplitude that corresponded to the 50% point on the psychometric curve and detection variability was defined as the difference in stimulus amplitude between the 75% and 50% point on the psychometric curve. For detection variability, larger differences between the two points was indicative of a flatter slope. Our main interest was whether detection threshold and detection variability changed when the object was moving toward the individual, as compared to when the object was not moving (i.e., Pre object movement). Therefore, for each participant, we calculated the relative detection threshold change and at each stimulation time by taking the ratio of the detection threshold at each stimulation time and the detection threshold at the Pre object movement stimulation time and multiplying that ratio by 100 (e.g., [Early detection threshold/Pre object movement detection threshold] × 100). Values below 100% are indicative of tactile facilitation compared to the Pre object movement baseline and values above 100% are indicative of tactile suppression compared to the Pre object movement baseline^5,24^. The same procedure was done to calculate relative detection variability change, in which the ratio of the variability at each stimulation time and the variability at the Pre object movement stimulation time was multiplied by 100.

To infer whether there was significant tactile modulation at each stimulation time, the relative detection threshold and detection variability changes were compared to 100% (i.e., representing the Pre object movement baseline perception), using one-sample t-tests. To infer whether the stimulation time influenced tactile modulation, the relative detection threshold and detection variability changes were submitted to separate 4 Stimulation Time (Early, Middle, Late-nc, Late) one-way RM analysis of variances. If the main effects for the one-way RM ANOVAs reached significance, post-hoc comparisons were decomposed using a Tukey’s HSD test and if sphericity was violated, a Greenhouse–Geisser correction was used to correct for degrees of freedom, with degrees of freedom reported to the 2nd decimal place. For all tests, alpha was set at *p* = 0.05.

## Results

**Figure 2:**
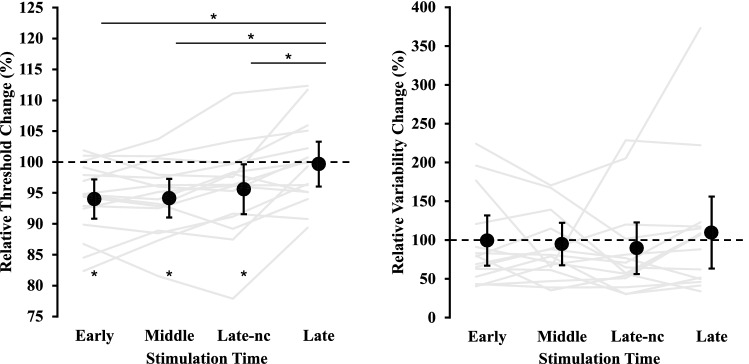
Left Panel: The statistically significant main effect of Stimulation Time (*p* < 0.01) for the relative threshold change, based on the Pre object movement condition. The stars on the bottom half of the graph represent a statistically significant difference from 100% at the Early, Middle and Late-nc stimulation times (all *p*’s < 0.03). The stars on the top half of the graph represent a statistically significant relative threshold change between the Late stimulation time and all other stimulation times (all *p*’s < 0.01). Right Panel: The relative variability change across Stimulation Time conditions (*p* = 0.52). Note that the detection variability difference was also relative to the Pre object movement condition. For both panels, grey lines represent individual participants, black dots represent mean values, and error bars represent 95% CIs.

The relative threshold change at the Early, Middle, and Late-nc stimulation times were all significantly lower than 100% (Early: *t* (15) = − 4.14, *p* < 0.01, 95% CI [− 9.05 − 2.90], *d* = 1.04; Middle: *t* (15) = − 4.10, *p* < 0.01, 95% CI [− 8.86 − 2.80], *d* = 1.03; Late-nc: *t* (15) = − 2.41, *p* = 0.03, 95% CI [− 8.34 − 0.50], *d* = 0.60), but there was no significant difference in the Late condition (*t* (15) = − 0.21, *p* = 0.84, 95% CI [− 3.88 3.20], *d* = 0.05; see Fig. 2). This result indicated that there was significant tactile facilitation in the Early, Middle and Late-nc condition but not the Late condition. Further, for relative threshold change, the RM ANOVA yielded a significant main effect of Stimulation Time (*F* (2.24, 33.52) = 4.11, *p* < 0.01, ${\eta }_{p}^{2}$ = 0.47). Post-hoc comparisons (HSD = 3.2%) revealed a significantly higher relative threshold change in the Late condition compared to all other conditions (i.e., Late–Early = 5.6%, Late–Middle = 5.5%, Late–Late-nc = 4.1%; see Fig. 2). This result indicated that the detection threshold was significantly higher in the Late condition compared to all other conditions. For detection variability, the relative variability change for all stimulation times] was not different from 100% (Early: *t* (15) = − 0.45, *p* = 0.97, 95% CI [− 32.1 30.8], *d* = 0.01; Middle: *t* (15) = − 0.41, *p* = 0.69, 95% CI [− 31.9 21.6], *d* = 0.10; Late-nc: *t* (15) = − 2.05, *p* = 0.50, 95% CI [− 42.8 21.8], *d* = 0.17; Late: *t* (15) = − 0.46, *p* = 0.66, 95% CI [− 35.3 54.5], *d* = 0.11) and the RM ANOVA did not yield a significant main effect of Stimulation Time (*F* (1.81, 27.18) = 0.76, *p* = 0.52, ${\eta }_{p}^{2}$ = 0.05). This result indicated that detection variability at each stimulation time did not differ from the Pre object movement baseline or each other.

## Discussion

The purpose of the current study was to investigate tactile processing while individuals anticipated tactile feedback from an object but without executing a reaching movement (i.e., during an object reception task). Importantly, we did not expect central and/or peripheral mechanisms to reduce the processing of tactile information because individuals did not make reaching movements toward the object. In line with our main hypothesis, tactile perceptual thresholds were decreased as the object approached the individual’s hand, compared to when the object was still (i.e., tactile facilitation). Further, tactile perceptual thresholds were reduced compared to baseline even when the object stopped unpredictably and did not make contact with an individual’s finger and thumb. Thus, this aligns with our hypothesis that the prediction of object contact and utilization of tactile feedback drives the facilitation of tactile processing during a reception task. Lastly, the main effect of stimulation time on relative perceptual threshold differences may suggest that the modulation of tactile processing was dependent on the time/ distance of the object relative to the hand. However, the lack of a systematic effect in the Late condition only (i.e., when the stimulation was presented shortly before object contact) may be explained by peripheral backward masking processes.

Both early (11% of the object’s total displacement) and around the middle of the object’s movement (44% of the object’s total displacement), individuals’ perceptual thresholds were significantly reduced compared to when measured prior to object movement (i.e., tactile facilitation). It is first important to consider methodological differences between the reception task employed in the current study and studies that did not find evidence for facilitation. In the current study, individuals did not execute reaching movements toward the object, whereas in previous work, tactile detection was measured during the execution of a juggling task^42^ or when moving toward the predicted location of a basketball during a catching task^43^. Thus, central efferent processes^8,13^ and peripheral afferent masking^49^ could at least partially account for reduced tactile sensitivity during reception or catching in previous work. In the current work, however, participants remained still throughout the task and only made minimal movement around the time of stimulation (i.e., movements of about ~ 1 mm for the 200 ms surrounding stimulation). Therefore, with minimal movements at a very slow speed, it is less likely that central efferent and/or peripheral afferent masking contributed to tactile perception or at least contributed to a much lesser extent^27^. But, beyond a lack of tactile suppression, the current study showed novel evidence for tactile facilitation. This result suggests that tactile processing was enhanced in anticipation of object contact and the subsequent utilization of tactile feedback. Indeed, during the reception task, the object’s trajectory resembled biological motion (i.e., bell shaped velocity; see Fig. 1) but was exactly the same for 80% of the trials. Thus, individuals could have used visual information to easily predict when the object would make contact with their hand^37–40^. Such sensory facilitation in the current study aligns with previous work showing evidence for cortical proprioceptive facilitation in preparation for the utilization of proprioceptive feedback for anticipatory postural adjustments during locomotion^41^. Further, the current study measured tactile processing at the index finger which received contact from the moving object, a stimulus location which can be deemed task-relevant^19^. Thus, the current findings extend task-relevant effects showing a lack of or reduced suppression^5,20,21,23^ to include facilitation at a task-relevant location when no reaching execution is required. Overall, tactile processing can be enhanced in anticipation of tactile feedback, at least when removing the suppressive influences of reaching execution toward the object.

To determine whether it was indeed the anticipation and prediction of tactile feedback that contributed to the facilitation effect, the current study included a condition in which individuals only had the mere expectation of object contact. To that end, this expectation was created by including a higher proportion of trials in which the object travelled the full distance and contacted a participant’s fingers (i.e., 80% of trials) compared to trials in which the object only travelled 22 cm and did not contact a participant’s fingers (i.e., on 20% of the trials). The experimental condition was randomly assigned on each trial, and thus, a contact trial was four times more likely to occur overall than a no-contact trial. Moreover, the stimulation was presented ~ 74 ms prior to object stopping on the no-contact trials. This provides further evidence that participants expected object contact at the time of stimulation. These trial proportions are consistent with previous work exploring the expectation to move and tactile processing. That is, participants were cued to move their right index finger on 80% of trials and an increased movement expectation was inferred by decreased reaction times when the right index finger was cued to move compared to the left index finger^12^. Although, it was not feasible to confirm a participant’s expectations in the current study, the timing of the stimulation—as well as previous work using similar trial structure—suggests that participants expected tactile feedback, even in the no-contact trials. When the stimulation was presented on these no-contact trials, the participant’s perceptual thresholds were reduced compared to the baseline condition, indicative of tactile facilitation. Further, the amplitude of facilitation was not significantly different from the early and middle stimulation times, trials which included object contact. Thus, the current results suggest that the mere expectation of object contact and the utilization of tactile feedback played a crucial role for tactile facilitation during this reception task (see also Ref.^50^ for another theoretical perspective on perceptual prediction and processing).

The current study also manipulated when the stimulus was presented relative to the start of object movement. When the stimulation was presented early or in the middle of the object’s trajectory, relative perceptual thresholds were lower than when it was presented near movement end. Further, perceptual thresholds in the Late condition were not different from the baseline condition (see Fig. 2). The lack of a facilitation or suppression effect may be explained by competing facilitatory processes, via the anticipation of tactile feedback, and suppressive processes, via backward masking. In the context of the reception task, the tactile feedback from the object contacting the index finger may have masked the perception of the weak electrical stimulus presented to the same index finger. This assertion is also supported by reduced relative perceptual thresholds in the Late-nc compared to the Late condition. The electrical stimulation was presented at the exact same time and point in the object’s trajectory (see Fig. 1). The main difference between the conditions was object contact in the Late and not the Late-nc condition, but it also worth considering differences in visual feedback. That is, the dowel visually stopped abruptly and unexpectedly only ~ 74 ms after the tactile stimulation was presented in the Late-nc condition. It can be argued that this visual cue presented shortly after the tactile stimulus could have masked or averted attention away from the tactile stimulus. But the results suggest that any attentional/ masking effect from the additional visual cue did not significantly alter perception as the Late-nc condition was associated with better perceptual performance than the Late and baseline conditions. Rather, the results suggest that object contact in close temporal proximity to the presentation of the electrical stimulus may have had a masking influence, even when the object contact occurred after the presentation of the electrical stimulus. It is also important to consider the time difference between the masking stimulus and the test stimulus. In the Late condition, the electrical stimulus was presented approximately 194 ms prior to object contact. In relation to other behavioural studies employing passive movement, reduced perception has been found up to 100 ms prior to movement onset. Further, when a participant’s opposite hand is the target, tactile facilitation at the static, target hand occurred approximately 350 ms prior to finger contact but was reduced approximately 250 ms prior to finger contact^33^. Thus, the current results may provide additional evidence for peripheral masking effects as early as 194 ms prior to a masking event.

When considering the influence of time or distance relative to object contact on tactile processing, we can compare relative perceptual threshold differences at the Early, Middle and Late-nc stimulation times. Indeed, peripheral masking likely had little to no influence on these conditions. When making these comparisons, there was no difference in the amplitude of tactile facilitation. This result suggests that the time or distance relative to object contact did not significantly influence tactile processing. Such a result also contrasts with the literature employing reaching actions and in which sensorimotor responses were altered over the course of the movement^33,45,51^. In the current study, however, no reaching movements were employed. Thus, it may be that within the constraints of the current task, tactile processing was facilitated for the entire trajectory of the object. That is, within ~ 700 ms or 26 cm of an object approaching an individual, tactile processing was facilitated the whole time. For the purposes of the current study, we only used one object trajectory, but future work can manipulate the speed of the object movement to dissociate whether distance or time from contact is a key factor for facilitation (e.g., stimulating at the same distance relative to contact but with differing times to contact or vice versa). Future work can also employ different object movement distances to investigate whether there is a minimum distance or time from contact at which tactile facilitation will occur at the receiving hand.

Overall, the current study explored the relationship between tactile processing and acquiring objects without accompanying reaching movements. Rather, individuals kept their limb still, while an object travelled to and contacted their receiving hand. As opposed to suppression, perceptual thresholds were decreased compared to baseline, indicative of tactile facilitation as the object moved toward the individual’s hand. Furthermore, the facilitation observed in the current study resulted from the expectation of object contact and the associated tactile feedback.

## Supplementary Information

Supplementary Figure S1.

## Data Availability

The perceptual data from the reported experiment are available at https://figshare.com/s/eb74aad15512886fa79b.
