# Supplementary material for: Tactile facilitation during actual and mere expectation of object reception
Source: Sci Rep. 2022 Oct 20;12:17514. doi: 10.1038/s41598-022-22133-z (PMC9585022; doi:10.1038/s41598-022-22133-z)
Supplement: Supplementary file 1 — Supplementary Figure S1. [file 41598_2022_22133_MOESM1_ESM.docx]

**Figure S1.** Psychometric curves for each condition and for each participant. Note that the stimulus amplitude differs for each participant because each participants had their own custom range of stimulus amplitudes based on an estimate of their initial threshold.
